# Thromboprophylaxis in Intensive Care Unit Patients: A Literature Review

**DOI:** 10.7759/cureus.3341

**Published:** 2018-09-21

**Authors:** Amna Ejaz, Munis M Ahmed, Azka Tasleem, Muhammad Rafay Khan Niazi, Muhammad Farhan Ahsraf, Imama Ahmad, Asma Zakir, Awais Raza

**Affiliations:** 1 Internal Medicine, King Edward Medical University/Mayo Hospital, Lahore, PAK

**Keywords:** venous thromboembolism, thromboprophylaxis, prophylaxis, intensive care unit(icu)

## Abstract

Thromboembolism is a major complication in hospitalized patients. Intensive care unit (ICU) patients have a greater risk of thrombotic events due to additional risk factors such as immobilization, mechanical ventilation, and central catheters. The diagnosis and management of deep vein thrombosis (DVT) and pulmonary embolism (PE) in critically ill patients are challenging and these conditions are associated with high mortality. Medical thromboprophylaxis with low molecular weight heparin (LMWH) as well as unfractionated heparin (UFH) has been shown to reduce the incidence of thromboembolic events in such patients. For patients with high risk of bleeding, mechanical thromboprophylaxis can be used. Literature database was conducted on Medline for articles published up to 2018 using particular search terms such as thromboprophylaxis and venous thromboembolism in ICU patients. The following review summarizes the existing data regarding thromboprophylaxis in ICU patients with special consideration to the use of mechanical prophylaxis and pharmacologic prophylaxis using heparin products.

## Introduction and background

Venous thromboembolism (VTE) is a condition that involves the formation of clots in the deep veins, particularly in the veins of the lower limb. This causes obstruction to blood flow resulting in symptoms like pain, swelling and discoloration [1]. The most common complication of venous thrombosis is the migration of these clots into other blood vessels, called embolism, particularly pulmonary embolism. According to research, half of the hospitalized patients are at a risk of thromboembolism [2]. The rate of VTE ranges from 10% to 80% percent in patients, who are not being given any prophylaxis [3-4]. Studies have proven the rate of VTE is greater in hospitalized than community patients [5].

The impact of thromboprophylaxis can be ascertained from the fact that it reduces the rate of thromboembolism in both medical and surgical patients. However, it decreases the mortality rate in surgical patients only, having little or no impact on the mortality rate among medical patients [6-7]. Thromboprophylaxis is of two varieties, primary and secondary. Primary prophylaxis is the one which is given to prevent the occurrence of deep vein thrombosis (DVT), which includes pharmacologic therapy like unfractionated heparin (UFH), low molecular weight heparin (LMWH), fondaparinux or mechanical therapy like pneumatic and graduated compression stockings [8]. Secondary prophylaxis involves early detection and management of venous thrombosis. The method of primary prophylaxis is determined by factors like the risk of thrombosis and hemorrhage, nature of the illness, the policy of the institution, cost and preferences. These factors help to classify patients into low, moderate and high-risk categories, each having a different method of prophylaxis. Moreover, the duration of prophylactic treatment differs from patient to patient depending on the risk classification.

Patients admitted to intensive care units (ICUs) are rated as high-risk patients even if they are being given prophylaxis [9-10]. Trials comparing the efficacy of pharmacological and mechanical thromboprophylaxis in ICU patients are but few. There is a need for further study regarding proper thromboprophylaxis in these patients in order to reduce mortality. Our study aims to review the literature from the past five years regarding the incidence, diagnosis, and prevention of VTE in ICU patients.

## Review

Epidemiology

DVT is prevalent in ICUs, particularly in Western countries. Asian countries have lower incidence comparatively. In a study in Thai surgical ICU patients, the incidence of DVT was found to be 3.6% which is comparable to a study in Tehran in which the incidence of DVT in ICU patients was found to be 3.5 % [11]. Two independent risk factors of DVT development are longer ICU stay and older age [12]. Another study was conducted in Chinese cancer patients admitted to ICU for the purpose of detecting VTE. It revealed a low incidence of VTE [13]. The incidence of VTE was 37.2% in patients with sepsis and septic shock [14]. Studies showed comparable results in adolescents [15].

Risk factors

Risk factors for thromboembolism can be divided into two groups, genetic and acquired. Genetic risk factors include loss and gain of coagulation function disorders [16]. According to studies, people having factor V Leiden or prothrombin 21210 mutation have the higher risk of thromboembolism than those without them [17]. Acquired risk factors include bed rest, age, hematologic cancers, immobilization, obesity, pregnancy, smoking, stroke, long-distance travel and certain inflammatory conditions [18]. The risk of VTE is higher in patients admitted to ICU than others, due to the higher number of risk factors specific to ICUs. Sepsis, vasopressor use, central catheters, mechanical ventilators, respiratory, cardiac or renal failure are common ICU related risk factors of VTE [19]. Research proves that patients with inserted catheters are at a three-fold greater risk for developing thromboembolism than those without them [20] Similarly, the duration of mechanical ventilation has an impact on the incidence of thromboembolism [14] Also, according to a recent study, the risk of developing thrombosis is directly proportional to the number of packed red blood cell (PRBC) transfusions [21].

Diagnosis

The diagnosis of VTE is highly dependent on the risk factors in a particular patient and the clinical presentation of the patient, for example, swelling, tenderness, hemoptysis, dyspnea etc. Also, different scoring systems help in diagnosis, most commonly employed for DVT is Well’s criteria [22]. Initially, following the criteria, patients were classified into high, intermediate and low-risk groups. Currently, Doppler venous ultrasound is the best imaging modality for diagnosis of DVT [23]. Others like contrast venography and magnetic resonance venography are also being employed, but they have certain limitations [24]. The diagnosis of pulmonary embolism (PE) also begins with the pre-test probability which is determined by Wells score, classified into low and high probability [25]. computed tomography pulmonary angiography (CTPA) is the gold standard for diagnosing PE [26].

The rationale for thromboprophylaxis

DVT and PE are very common complications in critical patients, especially those in ICU. Use of thromboprophylaxis can reduce mortality in such patients. Three randomized controlled trials (RCTs) held in ICU patients concluded that the incidence of DVT was significantly lower in the thromboprophylaxis group in comparison to the control group, irrespective of the type of thromboprophylaxis used [27-29]. According to current guidelines for DVT prevention in critically ill medical patients, either UFH or LMWH may be used. The Prophylaxis of Thromboembolism in Critical Care Trial (PROTECT) is a randomized clinical trial that is being held presently to study these different modes of thromboprophylaxis [8]. The protocol according to the American College of Chest Physicians (ACCP) guidelines is as follows: 1) Routine evaluation for VTE risk and thromboprophylaxis is recommended for critically ill patients (Grade 1A). 2) LMWH or low-dose UFH thromboprophylaxis should be given to patients with moderate risk for VTE (e.g, medically ill or postoperative general surgery patients); (Grade 1A). 3) Mechanical thromboprophylaxis is considered best for critical care patients who are at greater risk for bleeding, at least, until the bleeding risk decreases (Grade 1A). When the high bleeding risk decreases, pharmacologic thromboprophylaxis should be substituted for or added to the mechanical thromboprophylaxis (Grade 1C) [8].

Pharmacological thromboprophylaxis

Pharmacological prophylaxis with heparin is recommended for patients at the time of admission to the ICU. Heparin should be discontinued temporarily in patients with active bleeding or severe (<50,000/cc) thrombocytopenia. Though different thromboprophylaxis regimens have been suggested there is still no agreement based on evidence [30]. Until now, the PROTECT study comprising 3764 patients is the only RCT to have compared UFH with LMWH as VTE prophylaxis in the ICU. It did not include patients who were at higher risk for bleeding [31]. The patients were randomly assigned to two different groups. One group was given 5,000 IU of subcutaneous dalteparin once daily plus placebo once daily and the other received 5,000 IU of subcutaneous UFH twice daily. However, the difference between the incidence of proximal DVT in the dalteparin vs UFH group was not significant; (5.1% vs 5.8%, p=0.57). However, the rate of PE was significantly lower in the dalteparin group (1.3 %) compared with the UFH group (2.3 %) (p = 0.01). A recent meta-analysis by Park J et al. showed a significant reduction in the risk of DVT with heparin compared with the control group (LMWH: OR, 0.38; UFH: OR, 0.45), which was comparable to the previous review. However, on comparing UFH and LMWH, the efficacy was found to be comparable, which was also in agreement with the previous analysis [32-33]. Throm­boprophylaxis in critically ill patients may lead to bleeding which can be fatal. Moreover, both bleeding and the resulting discontinuation of thromboprophylaxis has a negative impact on clinical outcomes in the ICU. The meta-analysis also concluded that the differ­ence in major bleeding risk between UFH and LMWH was not significant [32].

According to the results of a retrospective observational cohort study, males having a body mass index ≥ 40 kg/m^2^ have the greater risk of developing VTE compared to females. However, in patients who were given a standard dose UFH, morbid obesity did not increase VTE risk overall. Also, morbid obesity was more likely associated with greater hospital and ICU length of stay [34].

A study comparing two types of LMWHs, enoxaparin and bemiparin showed that bemiparin was better than enoxaparin as a prophylactic anticoagulant for VTE in critically ill patients. It was associated with fewer local complications at the injection site. DVT was found in only 4% of the patients in the bemiparin group while 20% of the patients taking enoxaparin developed DVT. Confirmed PE was found in 14% of patients in the enoxaparin group as compared to no recorded case in the bemiparin group [35].

Anti-factor Xa levels can be used to clinically measure the effectiveness of LMWH anticoagulant; levels of 0.1 to 0.3 UI/ml are considered as adequate. Proper VTE prophylaxis is crucial in trauma patients with fractures of the lower extremity and pelvis. One such study explored whether dosing prophylactic enoxaparin using anti-Xa trough levels affected the incidence of VTE in such trauma patients. The study showed that in the majority of patients (84.5%) who had anti-Xa trough levels measured, the initial enoxaparin dose being given was sub prophylactic. Patients who were given enoxaparin according to anti-Xa trough level had a significantly lower risk of VTE compared to those in which anti-Xa levels were not measured; (1.7% v. 13.9%, p = 0.03) [36]. Since LMWHs are mostly excreted by the kidneys, they can accumulate in patients with renal insufficiency and thus have increased the risk of causing bleeding. Further, the risk of acute renal failure is higher in critically ill patients; at ICU admission, nearly one-third of patients have a creatinine clearance below 30 ml/minute [37]. A first meta-analysis failed to demonstrate the bio-accumulation of LMWH in critically ill patients with renal insufficiency [38]. Critical patients with acute kidney injury present a challenge in the provision of thromboprophylaxis as the dose has to be balanced with the risk for bleeding. A trial held in Denmark showed that the existing recommendation of 40 mg enoxaparin is not sufficient in patients with renal dysfunction and that 1 mg/kg is both safe and effective for thromboprophylaxis [39]. Another study consisting of a subgroup analysis of PROTECT was held in order to find out the safety and efficacy of LMWH VTE prophylaxis in critically ill patients with abnormal renal function. When dalteparin 5000 IU daily was compared with UFH 5000 IU twice daily in patients with renal insufficiency or end-stage renal disease (ESRD), there was no significant difference in the incidence of VTE or major bleeding. Patients with severe renal dysfunction who were on dalteparin did have a greater incidence of proximal DVTs compared to those on UFH; there was no greater risk of VTE or major bleeding [40].

Factor Xa inhibitors can also act as anti-coagulants by inhibiting the coagulation factor X and thus preventing clotting. In addition to heparin, their role in VTE prophylaxis should also be investigated in medically ill patients. In a case report in Pakistan, a patient was admitted to the medical ICU with hospital-acquired pneumonia. She had a low risk of VTE and so, she was advised to simply continue her rivaroxaban therapy-which she was already taking for avalvular atrial fibrillation- in addition to mechanical measures to prevent VTE. Unfortunately, she developed pulmonary VTE while she was being mechanically ventilated. This is the first case report of an incident in which a patient developed VTE despite being adequately anticoagulated with rivaroxaban [41]. Since VTE can still occur sometimes despite thromboprophylaxis with heparin, there is a need to study other agents for thromboprophylaxis. Aspirin has been shown to decrease the risk of VTE in surgical and high-risk medical patients but its effects in mechanically ventilated ICU patients are unknown. A study designed to investigate the effect of aspirin on thromboembolic events in mechanically ventilated patients showed a significant reduction in the odds of finding DVT with aspirin (OR 0.39, 95% CI 0.16–0.94; p = 0.036) [42]. So, aspirin may be helpful in preventing DVT in such patients.

Mechanical thromboprophylaxis

When anticoagulation is contraindicated, mechanical thromboprophylaxis using either graduated compression stockings (GCS) or intermittent pneumatic compression (IPC) may be proposed. Thromboprophylaxis by mechanical means alone is recommended for critical care patients at high risk of bleeding, in whom anticoagulants are contra-indicated [8]. According to one study, the use of IPC but not GCS was associated with a significantly lower VTE risk. No real association was found between the mechanical thromboprophylaxis and the type of prophylactic heparin used, recent trauma or recent surgery [43]. Pressure injuries have been found to be a notable complication of GCS in surgical ICU patients [44]. A recent meta-analysis of 12 trials found a trend of re­duced DVT risk with IPC, compared to the control group, but the reduction was not statistically significant. This shows that even though IPC is being commonly used, the thromboprophylactic effi­cacy of IPC is still questionable [33]. A study in China explored the comprehension and practice of mechanical thromboprophylaxis in ICU medical staff. It found that approximately 52.30% of all surveyed medical staff often practiced mechanical thromboprophylaxis. However, 25% of the included staff had never heard of mechanical thromboprophylaxis [45]. There is a need to remove concerns regarding IPC and GCS and properly educate the staff as these measures could help decrease VTE incidence in ICUs and improve the prognosis of critically ill patients by increasing the use of mechanical thromboprophylaxis.

Thromboprophylaxis compliance

The term thromboprophylaxis compliance refers to the extent to which ACCP prophylaxis guidelines are followed while administering any type of prophylaxis (pharmacologic or mechanical). According to a study conducted on 472 patients to evaluate if prophylaxis was being given in the right manner, it was concluded that 54.9% of patients were not being given appropriate prophylaxis [46]. This included patients who had absolute indications for prophylaxis but were not given prophylaxis, patients who had no indications for prophylaxis but were given prophylaxis, and patients who received the incorrect type of prophylaxis. Another study involving 364 patients showed that 16% of the patients were not receiving thromboprophylaxis and 45% of patients were not receiving pharmacologic thromboprophylaxis. The most common reasons were recent bleeding or surgery, provision of mechanical prophylaxis and thrombocytopenia [47]. Strategies to improve thromboprophylaxis compliance include the education of physicians and electronic reminders [48].

## Conclusions

ICU patients are at greater risk for VTE due to additional ICU related risk factors. DVT and PE in these patients can be diagnosed with venous Doppler ultrasound and CTPA, respectively. Thromboprophylaxis in these patients poses a challenge. Pharmacologic thromboprophylaxis with all types of heparin has been proven to significantly help reduce VTE in ICU patients. The efficacy of UFH and LMWH has turned out to be comparable with no increased risk of major bleeding. Amongst LMWH, bemiparin has been shown to be superior to enoxaparin as a prophylactic anticoagulant. Dosing LMWH with anti-factor Xa levels could reduce the risk of VTE. However, the data to support the efficacy of mechanical thromboprophylaxis is not strong enough. The choice for the best method for thromboprophylaxis still needs further study and research.
